# Clinical Characterization of Patients With COVID-19 in Primary Care in Catalonia: Retrospective Observational Study

**DOI:** 10.2196/25452

**Published:** 2021-02-08

**Authors:** Miguel Angel Mayer, Josep Vidal-Alaball, Anna Puigdellívol-Sánchez, Francesc X Marín Gomez, Angela Leis, Jacobo Mendioroz Peña

**Affiliations:** 1 Research Programme on Biomedical Informatics Hospital del Mar Medical Research Institute Faculty of Health and Life Sciences, Universitat Pompeu Fabra Barcelona Spain; 2 Health Promotion in Rural Areas Research Group Gerència Territorial de la Catalunya Central Institut Català de la Salut Sant Fruitós de Bages Spain; 3 Unitat de Suport a la Recerca de la Catalunya Central Fundació Institut Universitari per a la recerca a l'Atenció Primària de Salut Jordi Gol i Gurina Sant Fruitós de Bages Spain; 4 Facultat de Medicina Universitat de Vic-Universitat Central de Catalunya Vic Spain; 5 CAP Anton de Borja Consorci Sanitari de Terrassa Terrassa Spain; 6 Unitat d’Anatomia Humana Facultat de Medicina Universitat de Barcelona Barcelona Spain; 7 COVID-19 Response Unit Department of Health Generalitat de Catalunya Barcelona Spain

**Keywords:** COVID-19, risk factors, primary health care, angiotensin-converting enzyme inhibitors, angiotensin II type 2 receptor blockers, risk, characteristic, retrospective, observational, Spain, mortality

## Abstract

**Background:**

The country of Spain has one of the highest incidences of COVID-19, with more than 1,000,000 cases as of the end of October 2020. Patients with a history of chronic conditions, obesity, and cancer are at greater risk from COVID-19; moreover, concerns surrounding the use of angiotensin-converting enzyme inhibitors (ACEIs) and angiotensin type II receptor blockers (ARBs) and its relationship to COVID-19 susceptibility have increased since the beginning of the pandemic.

**Objective:**

The objectives of this study were to compare the characteristics of patients diagnosed with COVID-19 to those of patients without COVID-19 in primary care; to determine the risk factors associated with the outcome of mortality; and to determine the potential influence of certain medications, such as ACEIs and ARBs, on the mortality of patients with COVID-19.

**Methods:**

An observational retrospective study of patients diagnosed with COVID-19 in the Catalan Central Region of Spain between March 1 and August 17, 2020, was conducted. The data were obtained from the Primary Care Services Information Technologies System of the Catalan Institute of Health in Barcelona, Spain.

**Results:**

The study population included 348,596 patients (aged >15 years) registered in the Primary Care Services Information Technologies System of the Catalan Central Region. The mean age of the patients was 49.53 years (SD 19.42), and 31.17% of the patients were aged ≥60 years. 175,484/348,596 patients (50.34%) were women. A total of 23,844/348,596 patients (6.84%) in the population studied were diagnosed with COVID-19 during the study period, and the most common clinical conditions of these patients were hypertension (5267 patients, 22.1%) and obesity (5181 patients, 21.7%). Overall, 2680/348,596 patients in the study population (0.77%) died during the study period. The number of deaths among patients without COVID-19 was 1825/324,752 (0.56%; mean age 80.6 years, SD 13.3), while among patients diagnosed with COVID-19, the number of deaths was 855/23,844 (3.58%; mean age 83.0 years, SD 10.80) with an OR of 6.58 (95% CI 6.06-7.15).

**Conclusions:**

We observed that women were more likely to contract COVID-19 than men. In addition, our study did not show that hypertension, obesity, or being treated with ACEIs or ARBs was linked to an increase in mortality in patients with COVID-19. Age is the main factor associated with mortality in patients infected with SARS-CoV-2.

## Introduction

A highly pathogenic coronavirus, SARS-CoV-2, was first described in Wuhan in late December 2019 and has since spread worldwide [1,2]. As a result, the second meeting of the International Health Regulations Emergency Committee was convened by the Director-General of the World Health Organization (WHO) regarding the outbreak of this coronavirus to declare it a public health emergency of international concern [3], and it was declared a pandemic on March 11, 2020 [4]. Spain, particularly some geographical areas such as the autonomous communities of Catalonia and Madrid, has been one of the countries most affected by COVID-19, with more than one million cases by the end of October 2020 [5].

Since the beginning of the pandemic, it has been clearly shown that COVID-19 disproportionately affects patients with a history of chronic conditions such as cardiovascular disease, chronic obstructive pulmonary disease (COPD), hypertension, and diabetes mellitus [6,7]. Obesity and cancer have also been linked to worse outcomes and prognosis for COVID-19 [8]. The relationship between smoking and the severity of COVID-19 infection is also controversial. At the beginning of the pandemic, studies pointed to a protective relationship between smoking and COVID-19 and suggested that nicotine could have a protective effect against COVID-19 due to its minor anti-inflammatory properties. This effect could even be more marked considering that it could be masked by smoking-related toxicity and cessation of smoking when patients are severely ill with COVID-19 [9-11]. The information linking smoking and protection against COVID-19 prompted the WHO to issue a warning that the severity of COVID-19 disease was higher among smokers [12]. Other studies have supported this statement, indicating that tobacco smoking increases the lung gene expression of angiotensin-converting enzyme II (ACE2) and therefore increases the severity of COVID-19 [13-16].

Although angiotensin-converting enzyme (ACE) and ACE2 are distinct enzymes with different mechanisms of action [17], ACE2 has been linked to susceptibility to COVID-19 as well as its severity, as the viral protein involved in cell entry (spike S protein) binds to ACE2 [17-19]. In rat hearts, angiotensin-converting enzyme inhibitors (ACEIs) and angiotensin-receptor blockers (ARBs) have been shown to increase the expression of ACE2 [20], and it has been suggested that these treatments can predispose patients to more serious SARS-CoV-2 infection and worse outcomes and mortality [17]. Other studies have postulated that ACEI has a protective role in COVID-19 and could even be used as a treatment to reduce lung complications resulting from the disease [21].

Although the burden of managing the COVID-19 pandemic initially fell on hospitals, this situation has gradually changed; primary care services are handling more cases, which is requiring substantial changes in the way primary care services are delivered to populations to manage the COVID-19 pandemic [22]. Patients are usually encouraged to request advice and speak first to a primary care physician by telephone or a video call to avoid in-person appointments and going to primary care settings. Countries recognized to have strong primary care systems, such as Spain, Belgium, and the United Kingdom, have experienced high COVID-19 mortality rates [23,24]. A recent study analyzing the prognostic factors of patients with COVID-19 in primary care was also conducted in Catalonia, including 322 consecutive patients with COVID-19. Being older, male sex, and autoimmune disease were the main predictors of admission to hospital or death [25]. Analysis of the characteristics of the population followed up in primary care and comparison between the characteristics of patients with and without COVID-19 can provide useful information for decision-making in pandemic situations.

The objectives of this study were (1) to compare the characteristics of adult patients diagnosed with COVID-19 compared to patients without COVID-19 in primary care; (2) to determine the risk factors for these patients associated with fatality as the outcome; and (3) to analyze the influence of taking medications such as ACEIs and ARBs on the mortality of patients with COVID-19.

## Methods

### Population

An observational retrospective study was conducted on patients diagnosed with COVID-19 living in Spain, specifically in the Catalan Central Region, from March 1 to August 17, 2020. The population in the region over the age of 15 years was included in the study, with 348,596 patients. Patients older than 15 years are cared for by family and community specialists in primary care in Spain. The population of the area, including patients younger than 16 years, was 415,000 at the time of the study. Among the patients included in the study, 6.8% (23,844/348,596) had been diagnosed with COVID-19.

The data were extracted from the computerized medical records of the Primary Care Services Information Technologies (IT) System of the Catalan Institute of Health in Barcelona, Spain. The Primary Care Services IT System contains primary care electronic health records (EHRs) for over 6 million people in Catalonia, covering more than 80% of the Catalan population [26]. This system applies an anonymization process to maintain complete confidentiality and privacy of these data, following the European General Data Protection Regulation 2016/679 of April 27 and the Spanish Organic Law 3/2018 on Data Protection and Guarantee of Digital Rights. Variables such as demographic characteristics (ie, age and sex) and diverse comorbidities, such as hypertension, diabetes, obesity, dyslipidemia, COPD, being a current smoker, heart failure, cerebrovascular disease, and ischemic heart disease, were studied. Patients being treated with ACEIs or ARBs were also considered. The different diagnoses of risk factors and COVID-19 were based on the International Statistical Classification of Diseases and Related Health Problems 10th Edition (ICD-10); the codes related to these diseases are registered in the EHR database. Specifically, the codes used were based on the WHO and the Spanish Ministry of Health codification recommendations: U07.1 COVID-19; B97.29: Other coronavirus as the cause of diseases classified elsewhere and B34.2: Coronavirus infection, unspecified. The complete list of codes used in the study is included in Multimedia Appendix 1. The death events of the patients were also obtained from this database, which records the exact moment a patient dies.

The study protocol was approved by the University Institute for Primary Care Research Jordi Gol Health Care Ethics Committee (Code 20/066-PCV).

### Statistical Analysis

Categorical variables are described using frequencies and percentages. Continuous variables are described with means and standard deviation. Proportions of categorical variables were compared using the Fisher exact test, and the sample *t* test or Wilcoxon rank-sum test were used in the case of continuous variables. Multiple logistic regression analysis for predicting binary outcomes from continuous and categorical variables was applied. Multivariate logistic regression models were applied for the comparison between groups (patients with and without COVID-19, and deceased and living patients with COVID-19), in which risk factors were adjusted for age and sex. The level of significance used was *P*<.05. The statistical analysis was conducted using R version 3.6.3 (R Project) and Jamovi version 1.2.24.0.

## Results

The study population included 348,596 patients over 15 years of age registered in the Primary Care Services IT System of the Central Catalan Region in Spain. The mean age of the patients was 49.53 years (SD 19.4), and 108,762/348,596 (31.2%) of the patients were aged ≥60 years. Overall, 175,484/348,596 patients (50.3%) were female. The most common comorbidities and clinical conditions were hypertension (75,699/348,596, 21.7%), dyslipidemia (71,424/348,596, 20.5%), and obesity (69,501/348,596, 19.9%). Regarding patients with hypertension, 22,771/75,699 (30.1%) of them were being treated with ACEIs and 9487/75,699 (12.5%) were being treated with ARBs. A comparison of the demographics and risk factors of patients without COVID-19 and patients diagnosed with COVID-19 is shown in Table 1.

The characteristics of patients with and without COVID-19 are shown in Table 1. A total of 23,844 patients in the study population of 348,596 (6.8%) were diagnosed with COVID-19 during the study period, with a mean age of 49.9 years (SD 20.0) and no significant differences compared to patients without COVID-19 (*χ*^2^_1_=18.1, *P*=.10) adjusted for sex. However, COVID-19 diagnosis was more frequent in patients between 31 and 40 years of age, between 41 and 50 years of age, between 81 and 90 years of age, and aged ≥90 years, with significant differences between the groups with and without COVID-19 (*χ*^2^_7_=1521.5, *P*<.001). Of the 23,844 patients diagnosed with COVID-19, 13,763 (57.7%) were women. The most frequent clinical conditions among these 23,844 patients were hypertension (5267 patients, 22.1%), obesity (5181 patients, 21.7%) and dyslipidemia (4749 patients, 19.9%). Among the 5267 patients with COVID-19 and hypertension, 1701 (32.3%) were in treatment with ACEIs, and 729 (13.8%) were in treatment with ARBs.

Patients who had been diagnosed with COVID-19 were more likely to have diverse comorbidities. Diabetes (*P*=.01), obesity (*P*<.001), COPD (*P*=.001), cancer (*P*<.001), smoking (*P*<.001), heart failure (*P*<.001), and cerebrovascular disease (*P*<.001) were significantly more frequent among patients with COVID-19 compared to patients without COVID-19. Moreover, hypertension (*P*=.77) and ischemic heart disease (*P*=.02) were more frequent among patients with COVID-19; however, the differences were significant only in the case of ischemic heart disease. Finally, dyslipidemia (*P*<.001) was more frequent among patients without COVID-19, with a significant difference.

**Table 1:** Demographics and comorbidities of the study population, including patients with and without COVID-19 (N=348,596). Multivariate analysis of the risk factors was performed. Age was adjusted for sex and sex was adjusted for age. The remaining risk factors were adjusted for age and sex.

Demographics and risk factors			Patients with COVID-19 (n=23,844)	Patients without COVID-19 (n=324,752)	Adjusted odds ratio (95% CI)		*P* value	
**Demographics**								
	Age (years), mean (SD)		49.93 (19.4)	49.53 (20.0)	1.0 (0.99-1.0)		.10	
	**Age group (years), n (%)**					N/A^a^		<.001
		16-30	4239 (19.2)	61,382 (20.4)				
		31-40	4182 (18.9)	49,610 (16.5)				
		41-50	4381 (19.8)	56,457 (18.7)				
		51-60	3685 (16.6)	48,309 (16.0)				
		61-70	1841 (8.3)	39,163 (13.0)				
		71-80	1335 (6.0)	25,999 (8.6)				
		81-90	1763 (8.0)	16,884 (5.6)				
		>90	739 (3.3)	3620 (1.2)				
	**Sex**					0.73 (0.71-0.75)		<.001
		Female	13,763 (57.7)	161,721 (49.8)				
		Male	10,081 (42.3)	163,031 (51.2)				
**Risk factors**								
	Hypertension		5267 (22.1)	70,432 (21.7)	1.01 (0.97-1.04)		.77	
	Diabetes		2101 (8.8)	27,379 (8.4)	1.06 (1.01-1.12)		.01	
	Dyslipidemia		4749 (19.9)	66,675 (20.5)	0.94 (0.90-0.97)		<.001	
	Obesity		5181 (21.7)	64,320 (19.8)	1.10 (1.06-1.14)		<.001	
	Chronic obstructive pulmonary disease		685 (2.9)	8193 (2.5)	1.23 (1.14-1.34)		.001	
	Cancer		1765 (7.4)	21,785 (6.7)	1.09 (1.04-1.15)		<.001	
	Current smoker		3578 (15.0)	46,659 (14.3)	1.09 (1.05-1.13)		<.001	
	Heart failure		515 (2.2)	4301 (1.3)	1.63 (1.48-1.79)		<.001	
	Cerebrovascular disease		176 (0.7)	1658 (0.5)	1.48 (1.26-1.72)		<.001	
	Ischemic heart disease		627 (2.6)	8122 (2.5)	1.11 (1.02-1.20)		.02	

^a^N/A: not applicable.

A total of 2680/348,596 patients (0.77%) in the overall study population died during the study period. The number of deaths among patients without COVID-19 was 1825/324,752 (0.56%); meanwhile, the number of deaths among patients diagnosed with COVID-19 was 855 (3.72%), with an odds ratio (OR) of 6.58 (*χ*^2^_1_=2658.4, *P*<.001, 95% CI 6.06-7.15). However, the number of deaths among patients aged ≥60 years without COVID-19 was 1681 (1.67%), and the number of deaths among patients with COVID-19 was 828 (14.02%), with an OR of 8.36 (*χ*^2^_1_=3168.5, *P*<.001, 95% CI 7.66-9.12). Moreover, the number of deaths among patients aged <60 years without COVID-19 was 144 (0.07%), and that among patients diagnosed with COVID-19 was 17 (0.16%), with an OR of 2.44 (*χ*^2^_1_=18.1, *P*<.001, 95% CI 1.62-3.69).

The frequency of risk factors and the statistical analysis of the association of the different risk factors with mortality adjusted for age and sex among patients diagnosed with COVID-19 are shown in Table 2. The mean age of patients with COVID-19 who died was 83 years (SD 10.80), with significant differences compared to the mean age (48.7 years, SD 19.22) of patients who survived (*t*_1066.4_=–87.84, *P*<.001). Men with COVID-19 died more frequently than women compared to the group of patients who survived. The mean age of death in patients with COVID-19 (83.0 years, SD 10.80) was higher than the mean age of death in patients without COVID-19 (80.6 years, SD 13.3), with a significant difference (*t*_2015.5_=–4.9936, *P*<.001).

**Table 2:** Characteristics of deceased and living patients diagnosed with COVID-19.

Demographics and risk factors				Deceased patients with COVID-19 (n=855)		Living patients with COVID-19 (n=22,989)		Adjusted odds ratio (95% CI)			*P* value^a^	
**Demographics**												
	Age (years), mean (SD)			83 (10.8)		48.7 (19.2)		1.12 (1.11-1.13)			<.001	
	**Sex**								2.22 (1.90-2.60)			<.001
		Male	468 (54.7)		13,295 (57.8)							
		Female	387 (45.3)		9694 (42.2)							
**Risk factors**												
	Hypertension			587 (68.7)		4680 (20.4)		1.16 (0.98-1.37)			.09	
	Diabetes			278 (32.5)		1823 (7.9)		1.69 (1.43-1.99)			<.001	
	Dyslipidemia			383 (44.7)		4367 (19.0)		1.19 (1.03-1.39)			.03	
	Obesity			250 (29.2)		4931 (21.5)		1.08 (0.91-1.27)			.38	
	Chronic obstructive pulmonary disease			105 (12.3)		580 (2.5)		1.18 (0.92-1.51)			.19	
	Cancer			210 (24.6)		1555 (6.8)		1.19 (0.99-1.43)			.06	
	Current smoker			37 (4.3)		3541 (15.4)		0.98 (0.67-1.39)			.93	
	Heart failure			127 (14.9)		388 (1.7)		1.59 (1.26-1.99)			<.001	
	Cerebrovascular disease			21 (2.5)		155 (0.7)		0.84 (0.50-1.33)			.47	
	Ischemic heart disease			93 (10.9)		534 (2.3)		1.20 (0.93-1.54)			.15	
	Treatment with ACEIs^b^			152 (17.8)		1549 (6.7)		0.90 (0.74-1.09)			.32	
	Treatment with ARBs^c^			75 (8.8)		654 (2.8)		1.00 (0.76-1.30)			.98	

^a^In the multivariate analysis, sex was adjusted for age and age was adjusted for sex. The remaining risk factors were adjusted for age and sex.

^b^ACEIs: angiotensin-converting-enzyme inhibitors.

^c^ARBs: angiotensin II receptor blockers.

**Figure 1:**
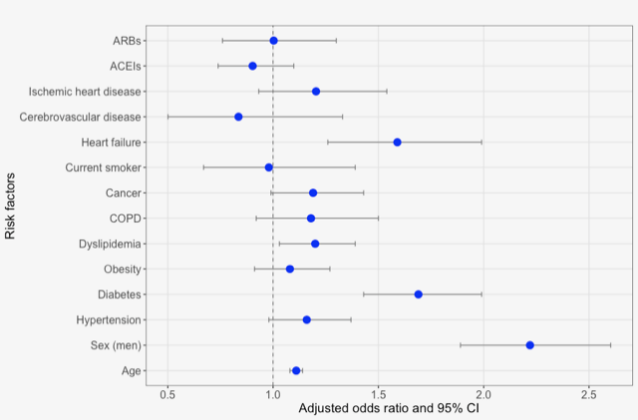
Risk factors associated with mortality in patients with COVID-19. The associations of each risk factor were estimated using logistic regression models adjusted for age and sex; sex was adjusted for age, and age was adjusted for sex. ACEIs: angiotensin-converting enzyme inhibitors; ARBs: angiotensin II receptor blockers; COPD: chronic obstructive pulmonary disease.

The logistic regression analysis showed that several conditions, such as diabetes, dyslipidemia, and heart failure, were associated with increased death risk with significant differences. Age and (male) sex were also associated with an increased death risk, with significant differences. Meanwhile, hypertension, obesity, COPD, cancer, being a current smoker, cerebrovascular disease, and ischemic heart disease did not show an increased risk of mortality among patients diagnosed with COVID-19. Figure 1 shows the ORs and 95% confidence intervals for the association between these risk factors and mortality described in Table 2. The use of ACEIs and ARBs for the treatment of hypertension, did not show an increased risk of mortality among patients diagnosed with COVID-19.

## Discussion

### Principal Findings

We report the clinical characteristics and mortality rates of 23,844 patients diagnosed with COVID-19 in primary care, who constituted 6.84% of the population studied in the Central Catalan Region aged ≥16 years. This is one of only a few larger studies of COVID-19 patients conducted in primary care settings [25,27,28] and the only one to compare the characteristics and risk factors of patients diagnosed with COVID-19 with those of other patients without COVID-19 in the same health care area. Although there is no significant difference in age between patients with and without COVID-19, age is the main factor associated with mortality in patients with COVID-19, along with male sex, diabetes, dyslipidemia and heart failure. On the other hand, although obesity is more frequent among patients with COVID-19, it did not show an increase in the risk of mortality in this study. Moreover, ACEI or ARB treatment did not show an increase in the risk of mortality.

In our study, we report lower rates of comorbidity among patients diagnosed with COVID-19 compared to another study carried out in Catalonia in primary care, with rates of 33.9% for hypertension, 14.3% for diabetes, 5.9% for COPD, and 11.5% for cancer. In contrast, they reported lower rates of dyslipidemia (13.7%) and obesity (14.3%) [25]. In this study, they reported a mean age of 56.7 years, more than 7 years older than the mean age in our study; this aspect could explain the differences in the frequency of the mentioned comorbidities.

Numerous studies have shown that male sex and older age are associated with higher COVID-19–related mortality, a conclusion which our study strongly supports [14,29,30]. Although studies based on hospital data have found that the frequency of SARS-CoV-2 infection was higher in men [2,31-35], we found that women more frequently contracted COVID-19. Regarding mortality and age, we report that the death rate among patients aged ≥60 years with COVID-19 was 8.36 times higher than that among patients aged ≥60 years without COVID-19, with age being a strong predictor of mortality among people diagnosed with COVID-19, in particular those older than a certain age (≥60 years).

In our study, diabetes was more common in patients with COVID-19 and was associated with an increased risk of death. This finding is in accordance with a meta-analysis that analyzed the association between comorbid diabetes and disease severity or death [35,36]. As shown in our study, diabetes is one of the most prevalent comorbidities in patients with COVID-19, and there is a significant association with a greater severity of the illness or death in primary care; this association is in line with in-hospital mortality for patients with COVID-19, for which up to a 3-fold increased risk has been found [37]. Likewise, in our study, heart failure was more frequent among patients with COVID-19 and was associated with an increased risk of mortality. Other studies have reported similar results [30]. Although we found that patients with obesity were more common among those diagnosed with COVID-19, contrary to other studies, we did not find an increased risk of mortality in these patients [38]. In addition, surprisingly, hypertension, one of the most important factors reported as a mortality risk factor [31], and ischemic heart disease were neither more frequent nor associated with a higher mortality risk in our study.

As for smoking, we found that smokers were more frequently infected with COVID-19, although with no increased risk of mortality. Regarding risk factors such as cancer and cerebrovascular disease, patients diagnosed with COVID-19 were not at a higher risk of mortality. In addition, our results are in keeping with those showing that ACEIs and ARBs are not significantly associated with an increased risk of death among patients with COVID-19 [2,35,39] when adjusting for age and sex. Although the frequency of the different risk factors analyzed, except in the case of dyslipidemia, was higher among patients with COVID-19, this aspect should be considered as important to take into account in the management of these patients; we have seen that mortality is linked to some of these factors, such as diabetes, dyslipidemia, and heart failure. In addition, the health characteristics of the population followed up in primary care settings can be useful to have a better knowledge of

### Limitations

The study has several limitations. The diagnostics of COVID-19 registered in the Primary Care Services IT System during the early months of the pandemic were based on both clinical and polymerase chain reaction (PCR) testing. This is a pragmatic approach due to the fact that PCR testing was not fully available in primary care during the first few weeks of the pandemic. After the first months of pandemic the number of PCR tests performed increased, and for this reason the number of COVID-19 diagnoses may be underestimated during the period of our study. Although we included the majority of relevant risk factors and comorbidities associated with COVID-19 in the study, additional conditions should be considered, which may have an impact on the analysis and its interpretation. Although the coverage of patients followed up by primary care physicians is approximately 80% of the population in Catalonia, including this health care area studied, it is unlikely but possible that the remaining 20% of patients have other demographic or health characteristics that could affect the results. In addition, as can occur in other diseases, the registration and mortality of cases can be underestimated or can be affected by factors such as gender; this may modify some of the results presented.

### Conclusions

At the beginning of the COVID-19 outbreak, attention was focused on the characteristics of patients diagnosed with COVID-19 who were admitted to hospital. However, after the period of our study, the attention shifted to community and primary care services, coinciding with the work overload in primary care settings and the possibility of more extensive SARS-CoV-2 testing. Our study is focused on patients in primary care with COVID-19, unlike most previous studies on COVID-19, which are based on hospital data. As we observed in our study, hypertension, one of the risk factors associated with COVID-19, was neither more frequent nor associated with higher mortality in patients with COVID-19 in primary care. We observed that women were more affected by COVID-19, unlike the majority of studies, which reported that men more frequently contracted the disease. In addition, our study did not find an associated risk between obesity and COVID-19, another risk factor associated with increased COVID-19–related mortality. Furthermore, treatment with ACEIs or ARBs was not associated with a higher mortality rate among patients infected with SARS-CoV-2. Age was the most important factor associated with mortality in patients with COVID-19.

Further studies focused on community and primary care are needed to provide new insights into SARS-CoV-2 infection and how to address outbreaks and improve health care strategies in pandemic situations.

## Appendix

Multimedia Appendix 1 Full list of diagnosis codes used in the study.

## Abbreviations

ACE: angiotensin-converting enzyme
ACE2: angiotensin-converting enzyme II
ACEI: angiotensin-converting enzyme inhibitor
ARB: angiotensin type II receptor blocker
COPD: chronic obstructive pulmonary disease
EHR: electronic health record
ICD-10: International Statistical Classification of Diseases and Related Health Problems 10th Edition
IT: information technology
OR: odds ratio
PCR: polymerase chain reaction
WHO: World Health Organization
