# Supplementary material for: Clinical Characterization of Patients With COVID-19 in Primary Care in Catalonia: Retrospective Observational Study
Source: JMIR Public Health Surveill. 2021 Feb 8;7(2):e25452. doi: 10.2196/25452 (PMC7871981; doi:10.2196/25452)
Supplement: Multimedia Appendix 1 [file publichealth_v7i2e25452_app1.docx]

**Full list of diagnoses codes used in the study**

| Diagnosis | Codes in ICD10 |
| --- | --- |
| COVID-19, virus identified | U07.1 |
| Other coronavirus as the cause of diseases classified elsewhere | B97.29 |
| Coronavirus infection, unspecified site | B34.2 |
| Pneumonia due to SARS-associated coronavirus | J12.81 |
| SARS-associated coronavirus as the cause of diseases classified elsewhere | B97.21 |
| Nicotine dependence | F17 |
| Disorders of lipoprotein metabolism and other lipidemias | E78 |
| Diabetes mellitus | E08-E13 |
| Hypertensive diseases | I10-I16 |
| Chronic obstructive pulmonary disease | J40-J47 |
| Neoplasms | C00-C96, D00-D49 |
| Overweight and obesity | E66 |
| Heart failure | I50 |
| Ischemic heart diseases | I25 |
| Cerebrovascular diseases | I60-I69 |
